# Mass Cytometry and Single-Cell Transcriptome Analyses Reveal the Immune Cell Characteristics of Ulcerative Colitis

**DOI:** 10.3389/fmolb.2022.859645

**Published:** 2022-06-23

**Authors:** Yongxin Luo, Shiying Liu, Huibiao Li, Jiangtao Hou, Wenjia Lin, Zewen Xu, Tianyu Lu, Yanwu Li, Bin Peng, Shijing Zhang, Xue Han, Zuoliang Kuang, Yi Wen, Jiazhong Cai, Fengbin Liu, Xin-Lin Chen

**Affiliations:** ^1^ School of Basic Medical Sciences, Guangzhou University of Chinese Medicine, Guangzhou, China; ^2^ The First Affiliated Hospital, Guangzhou University of Chinese Medicine, Guangzhou, China; ^3^ Science and Technology Innovation Center, Guangzhou University of Chinese Medicine, Guangzhou, China; ^4^ Pi-Wei Institute, Guangzhou University of Chinese Medicine, Guangzhou, China; ^5^ The First Clinical College, Guangzhou University of Chinese Medicine, Guangzhou, China

**Keywords:** ulcerative colitis, CyTOF, scRNA-seq analysis, immune cells, Tregs

## Abstract

**Background:** The pathogenesis of ulcerative colitis (UC) is closely related to immunity. The immune characteristic differences between active UC (UCa) and inactive UC (UCin) have not been completely explained. Mass cytometry (CyTOF) and single-cell RNA sequencing (scRNA-seq) were used to analyze the immune cells of UCa, UCin and healthy control (HC) subjects to determine the specific immune characteristics.

**Methods:** The immune cell subsets among UCa, UCin, HC were distinguished using CyTOF analysis. scRNA-seq analysis was used to validate the results of CyTOF. Gene ontology (GO) and Kyoto Encyclopedia of Genes and Genomes (KEGG) pathway analyses were performed to understand the roles of differential immune cell subsets.

**Results:** After CyTOF analysis and validation of scRNA-seq analysis, differential immune cell subsets mainly contained TNF^+^IL^-^17A^++^ effector memory (EM) Tregs, CXCR3^+^CTLA4^+^ EM Tregs, CXCR3^++^CCR7^+^ B cells, HLA-DR^+^CCR7^+^ dendritic cells (DCs) and CTLA-4^+^ natural killer (NK) cells. In comparison to HC, CCR6^+^TNF^+^CD161^+^ EM T cells were highly enriched in UCa and UCin. Besides, UCa was characterized by an increase in CD38^+^TNF^+^ EM Tregs, CXCR3^+^CCR4^+^ naïve B cells, HLA-DR^+^CD14^+^IL21^+^ macrophages/monocytes, HLA-DR^+^CCR7^+^ DCs, AHR^+^CD14^+^ cytotoxic NK (cNK) cells and CD8A^+^IFNG^+^ cNK cells. Decreases in CD38^+^CD27^+^ plasmablasts, CXCR3^+^CD38^+^ regulatory NK cells, and CXCR3^+^CCR7^+^ tolerant NK cells in UCa were discovered.

**Conclusions:** Novel immune cell subsets which was used to distinguish UCa, UCin and HC were identified. This information might be utilized to distinguish the patients with UCa and UCin.

## Introduction

Ulcerative colitis (UC) is an idiopathic chronic inflammatory disease, that is, mainly confined to the colorectal mucosa and submucosa (Ordas et al., 2012). UC comprises active stage (UCa) and inactive stage (UCin) disease. The main clinical manifestations in UCa are abdominal pain and bloody diarrhea, which sometimes can be accompanied by fever, loss of appetite, nausea and vomiting (Conrad et al., 2014). Patients with UCin usually have no obvious symptoms or may only have mild diarrhea or abdominal pain. For patients with UCin, the symptoms may be aggravated due to improper diet, fatigue, mental stimulation or other factors, and the disease may become active-stage disease. At present, the aim of treatments for UC is to alleviate mucosal inflammation and to induce and maintain remission of symptoms and disease. Local therapy with 5-aminosalicylate (5-ASA) is the treatment of choice to induce remission (Langan et al., 2007; Caselli et al., 2010; Kornbluth and Sachar, 2010).

The clinical process of UC is unpredictable and is characterized by alternating periods of active and inactive UC. Some studies have pointed out that these repetitive cycles of inflammation have been associated with the development of extensive dysplasia and eventual progression to malignancy (Sunkaran et al., 2011; Parray et al., 2012; Ungaro et al., 2019). Therefore, tailoring the clinical management of UCa and UCin patients and maintaining stable inactivity are of great significance for disease control and prognosis and the improvement in quality of life.

Previous studies have tried to explore the differences in gene expression between UCa and UCin patients. Zhao et al. conducted a gene expression study comparing intestinal biopsies of UCa and UCin patients and found that a major differential feature between them was the mobilization of marker genes and proteins for the epithelial-mesenchymal transition (EMT) pathway only in Uca patients (Zhao et al., 2015). Another study pointed out that the recovery of CD226^−^TIGHT^+^FoxP3^+^ and CD226^−^TIGIT^-^FoxP3^+^ regulatory T cells (Tregs) might be helpful for promoting clinical remission of patients with UCa (Long et al., 2020a). However, these findings do not completely explain the differences between them. Thus, it is necessary to further compare the differences.

As the most common and widely used cell quantitative analysis technique in molecular biology, cytometry by time of flight (CyTOF) has the advantages of high throughput and repeatability. CyTOF can analyze thousands of cells in a short time and can capture the various characteristics of each cell when combined with specific antibodies to select and analyze the target population rapidly and accurately (Spitzer and Nolan 2016). In the past few decades, its application has led to unprecedented achievements in research on the immune system and other fields of cell biology (McKinnon 2018).

In this study, to better guide the clinical diagnosis and management of UC in active and inactive stages, CyTOF and scRNA-seq analyses were used to analyze the different cells among patients with Uca and UCin and healthy control (HC) subjects to determine the specific immune characteristics of the disease.

## Materials and Methods

### Preprocessing of the Flow Cytometry Standard Files for CyTOF

The UC-related flow cytometry standard (FCS) files were preprocessed and then downloaded from Cytobank (https://www.cytobank.org/) (Chen and Kotecha 2014). The channel of each FCS file was scaled according to the minimum and maximum proportion to avoid the data deviating from the recognizable area. Then, the cell population was identified by manual gating. The positive or negative cell subsets of two visual markers were selected and further stratified until the target population in a series of marker combinations was captured. The gating subsets were captured in Cytobank, including T cells (CD45^+^CD3^+^ cells), Tregs (CD45^+^CD8a^−^CD4^+^CD25^+^CD127^-^ cells), B cells (CD45^+^CD3^−^CD19^+^ cells), innate immune cells (CD45^+^CD3^−^CD19^−^ cells) and NK cells (CD3^−^CD45^+^CD56^+^CD16^−^ cells). To ensure channel consistency, the CyTOF FCS files were uploaded to FlowJo software (FlowJo, Ashland, OR).

### CyTOF Data Analysis

All statistical calculations were conducted in R (version 4.0). The R packages employed in the analysis process included cytoWorkflow (Nowicka et al., 2017), CATALYST, flowCore (Hahne et al., 2009). FlowSOM (Van Gassen et al., 2015) was used for clustering, while t-stochastic neighbor embedding (t-SNE) (Kobak and Berens 2019; Zhou and Jin 2020) was used to reduce the dimensionality. Heatmaps were used to visualize differences in clusters, and boxplots were used to visualize differences in selected immune cell subsets (nodes). The formation of heatmaps and boxplots was accomplished using ggplot2 (https://cran.r-project.org/web/packages/gplots/index.html). Kruskal-Wallis test was used for comparison among multiple groups, and Wilconxon rank sum test was used for comparison between two groups, and *p* ≤ 0.05 was considered a statistically significant difference.

The detailed methods of visualization and calculation were as follows. 1) To visualize the differences in clusters, the proportion of 39 nodes was determined by the abundance of cluster cells. Analysis of variance of cell population abundance was used to compare the proportions of cell types to highlight populations with different proportions. 2) To visualize the differences in the selected nodes, the *p*-value of selected cell subsets was calculated, and the significant differences among them in individuals were compared using box line diagrams. 3) t-SNE dimension reduction of selected nodes and their important cytokines was used for revalidation.

### Data Acquisition of scRNA-Seq

To verify the CyTOF results, two datasets were collected from the GEO database (GSE125527 and GSE116222) (Boland et al., 2020; Mitsialis et al., 2020). Moreover, we also collected samples (colon tissues) for single-cell RNA sequencing to further verify the CyTOF results.

### Human Subjects

The Ethics Committee of The First Affiliated Hospital of Guangzhou University of Chinese Medicine approved the study (No: ZYYECK [2019]160). All the participants signed the informed consent form. The inclusion criteria were as follows: 1) participants were diagnosed with Uca or UCin or were healthy control subjects based on “The Asia-Pacific consensus on ulcerative colitis” published by APAGE on IBD in 2010 (Ooi et al., 2010); and 2) participants were aged between 18 and 75 years old. Then, all collected tissue samples will be sent to Huada Gene Technology Co., Ltd. (Shenzhen, China) for processing.

### Single-Cell Isolation

Colon tissues were placed in RPMI 1640 medium supplemented with 100 μg ml^−1^ streptomycin, 100 U ml^−1^ penicillin and 10 mm HEPES (Gibco, Gaithersburg, MD) on ice. Biopsies were thawed, washed twice in PBS and digested with collagenase type II (Worthington Biochem) under stirring (250 rpm) at 37°C for 20 min. The cell suspension was washed in PBS and passed through a 70-μM filter. Cells were counted with an automated cell counter, and their viability was confirmed with Trypan blue. Single cells were resuspended in freezing buffer (90% FBS and 10% DMSO freezing media), placed into a freezing container (Thermo Scientific Nalgene Mr Frosty) at −80°C, and then transferred to liquid nitrogen for long-term storage.

### 10X Genomics Library Preparation and Sequencing

Cellular suspensions were encapsulated in droplets using GemCode technology and processed by Huada Gene Technology Co., Ltd. (Shenzhen, China). In brief, gel beads with barcodes, UMIs (unique molecular identifiers), primers and enzymes were mixed with single cells by using a GemCode Single-Cell Instrument (10X Genomics, Pleasanton, CA, United States). In droplets, the cells broke, and the released mRNA was linked to the cell tag sequence on the gel beads to form a single-cell GEM structure (gel bead in emulsions). cDNA for sequencing on Illumina platforms was generated by reverse transcription using a GemCode Single-Cell 3’ Library Kit. Cell mRNA was reverse transcribed in droplets to form cDNA and then demulsified to construct indexed sequencing libraries. Sequencing libraries were then subjected to scRNA-seq using the Illumina platform.

### Data Analysis for scRNA-Seq

The Seurat package (3.2.2) was employed to merge and cluster datasets and to analyze differential gene expression (Satija et al., 2015). The entire work flow was as follows:

Quality control (QC) and merging Seurat objects: The QC process was performed using Seurat (version 3.2.2) (Butler et al., 2018). Single cells with the following characteristics were considered as low quality cells. The single cells are removed if they met the following criteria: 1) single cells containing less than 400 genes; 2) single cells with more than 4,000 genes; 3) single cells with more than 50% mitochondria-specific genes. For the GSE116222 database, the IntegrateData function was used to eliminate batch effects among the patients.

Dimensionality reduction: Scaled and log-normalized data were subsequently used to enable dimensionality reduction, and the main cell clusters were identified using the FindClusters function and visualized using t-SNE.

Cluster cell type annotation: To identify the markers of each cluster, the FindAllMarkers function was used. The main cell types were then recognized based on the markers obtained from the CellMarker database (Zhang et al., 2019). Eventually, T cells, B cells and NK cells were selected for further verification.

Verification of differential expression of specific factors: The variation in the expression of specific subsets in different clusters obtained by CyTOF analysis was visualized using a box graph. It was verified that these specific genes were veritably expressed in specific cell clusters. Differentially expressed gene-GO/KEGG analysis: To further investigate UC disease-associated cell functional states and potential molecular regulators, GO/KEGG analysis of differentially expressed genes (DEGs) in UC mucosa was conducted. Functional enrichment analysis was performed using the “ClusterProfiler” package in R software with a significant level of q-value < 0.05, for GO biological processes and KEGG pathways.

In addition, we also performed a differential abundance test using miloR (Dann et al., 2022), which will test for significant differences in cell abundance between the two different conditions, and the associated gene signatures.

## Results

The CyTOF data contained colonic mucosa from 18 UCa, 14 UCin and 18 HC subjects as well as peripheral blood from 22 UCa, 5 UCin and 25 HC subjects. Immune cell subsets were derived for dedicated analysis, and clusters with similar phenotypes were formed into branches of hierarchical clustering tree diagrams. Based on the direction of branch splitting, the tree nodes were labeled node X in the corresponding figures. The differences in immune cell characteristics among UCa, UCin, and HC subjects were distinguished by comparing the abundance of different cell populations.

### CCR6^+^TNF^+^CD161^+^ Effector Memory T Cells Are Enriched in Active Ulcerative Colitis Mucosa

**FIGURE 1:**
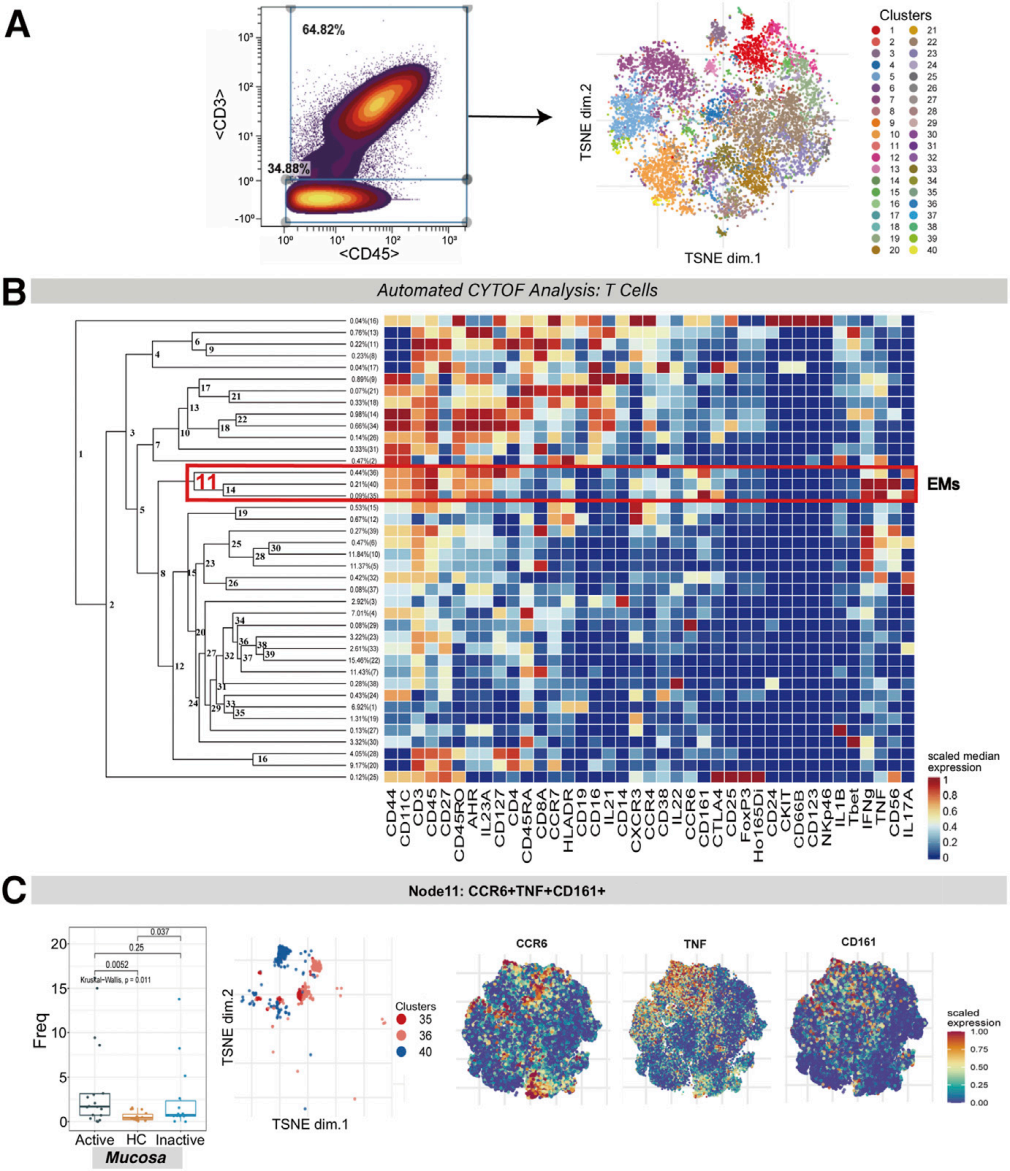
CyTOF analysis of the CCR6^+^TNF^+^CD161^+^ T cell subset in UC mucosa. **(A)** Gating steps for screening T cells that were then derived and used for dimensionality reduction (t-SNE) and clustering (FlowSOM) are shown on the left; the t-SNE plot of selected T cells is shown on the right. **(B)** Cluster dendrogram and heatmap of selected T cells. The branch numbers in the dendrogram corresponded to the cluster numbers in the t-SNE plot (from Panel 1A). The selected nodes highlighted in red are circled with red lines, and the subset names are labeled on the right. The heatmap represents the scaled value for the relative abundance of each marker per cluster. Red represents high expression, and blue represents low expression. **(C)** The abundance boxplot of node 11 among the three groups is shown on the left with the Kruskal-Wallis test results; statistical significance was set at *p* < 0.05; the t-SNE plot of node 11 is shown on the right, with CCR6/TNF/CD161 marker heatmaps for reference.

To discover the differences among T cell populations across the three groups, CD3^+^CD45^+^ T cells were clustered in a dedicated analysis (Figure 1A,B). Multiple T cell subsets mainly contained effector memory [EM] (CD45RA^−^CD45RO^+^CCR7^−^CD27^+/-^), central memory [CM] (CD45RA^−^CD45RO^+^CCR7^+^CD27^+^), and naïve (CD45RA^+^CD45RO^−^CCR7^+^CD27^+^) cells.

A population of EM T cells (node 11, containing clusters 35, 36 and 40) was found to express CCR6, TNF, CD161, IFNG and IL-17A. There was a statistically significant difference between the abundance levels of these cells among the UCa, UCin and HC mucosa groups (*p* = 0.011). These EM T cells were enriched in both UCa and UCin mucosa compared to HC mucosa (*p* < 0.05, Figure 1C). However, none of these differences were statistically significant among the three groups in peripheral blood.

### CD38^+^TNF^+^ Effector Memory Tregs Were Enriched in Active Ulcerative Colitis Mucosa While CD27^+^CXCR3^+^ Effector Memory Tregs Were Increased in Inactive Ulcerative Colitis Peripheral Blood

**FIGURE 2:**
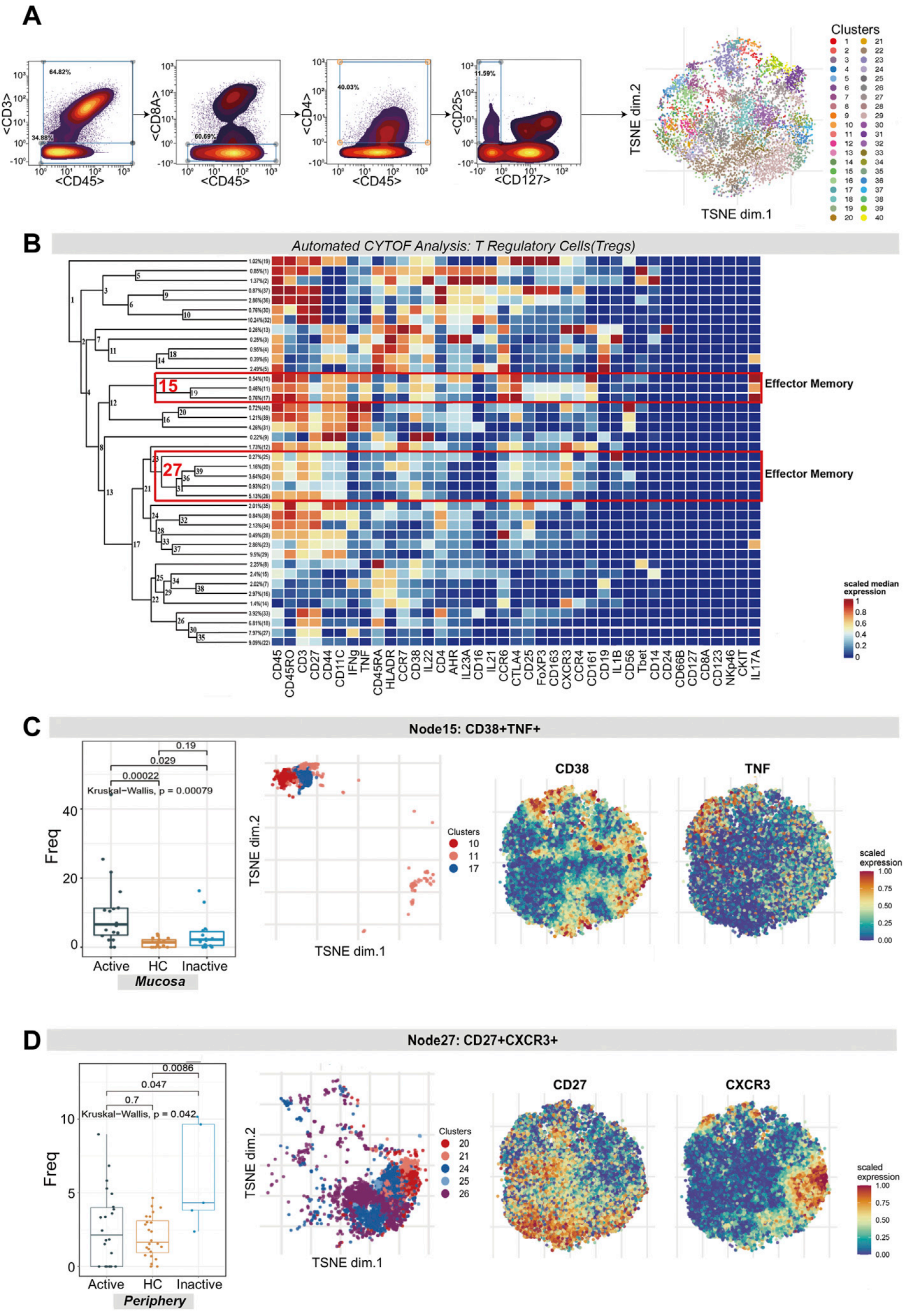
CyTOF analysis revealed the expression of two effector memory Treg subsets in UC patients and HCs. **(A)** Gating steps (left) and t-SNE plot (right) of selected Tregs. **(B)** Cluster dendrogram, heatmap and selected nodes of Tregs. **(C,D)** Abundance boxplots and t-SNE plots of node 15 **(C)** and node 27 **(D)** in Tregs, with respective selected marker heatmaps for reference.

Tregs were manually identified based on the expression of CD45, CD4, CD25, and CD127 and were then used for dedicated automated analysis (Figure 2A,B). Treg subsets were allocated on the basis of the same guidelines as for T cell subsets.

As shown in the figure, the abundance level of EM Tregs across node 15 (clusters 10, 11 and 17) and node 27 (clusters 25, 20, 24, 21 and 26) was statistically significant, with *p* = 0.00079 and *p* = 0.042, respectively. Node 15, which coexpressed CD38 and TNF, was comparatively enriched in Uca mucosa but decreased in HC and UCin mucosa (*p* < 0.05, Figure 2C). Node 27, which coexpressed CXCR3, CD27, CTLA4 and IL1B, was specifically increased in UCin peripheral blood compared with UCa and HC peripheral blood (*p* < 0.05, Figure 2D).

### CD38^+^CD27^+^ Plasmablasts Were Diminished Whereas CXCR3^+^CCR4^+^ Naïve B Cells Were Expanded in UCa Mucosa

**FIGURE 3:**
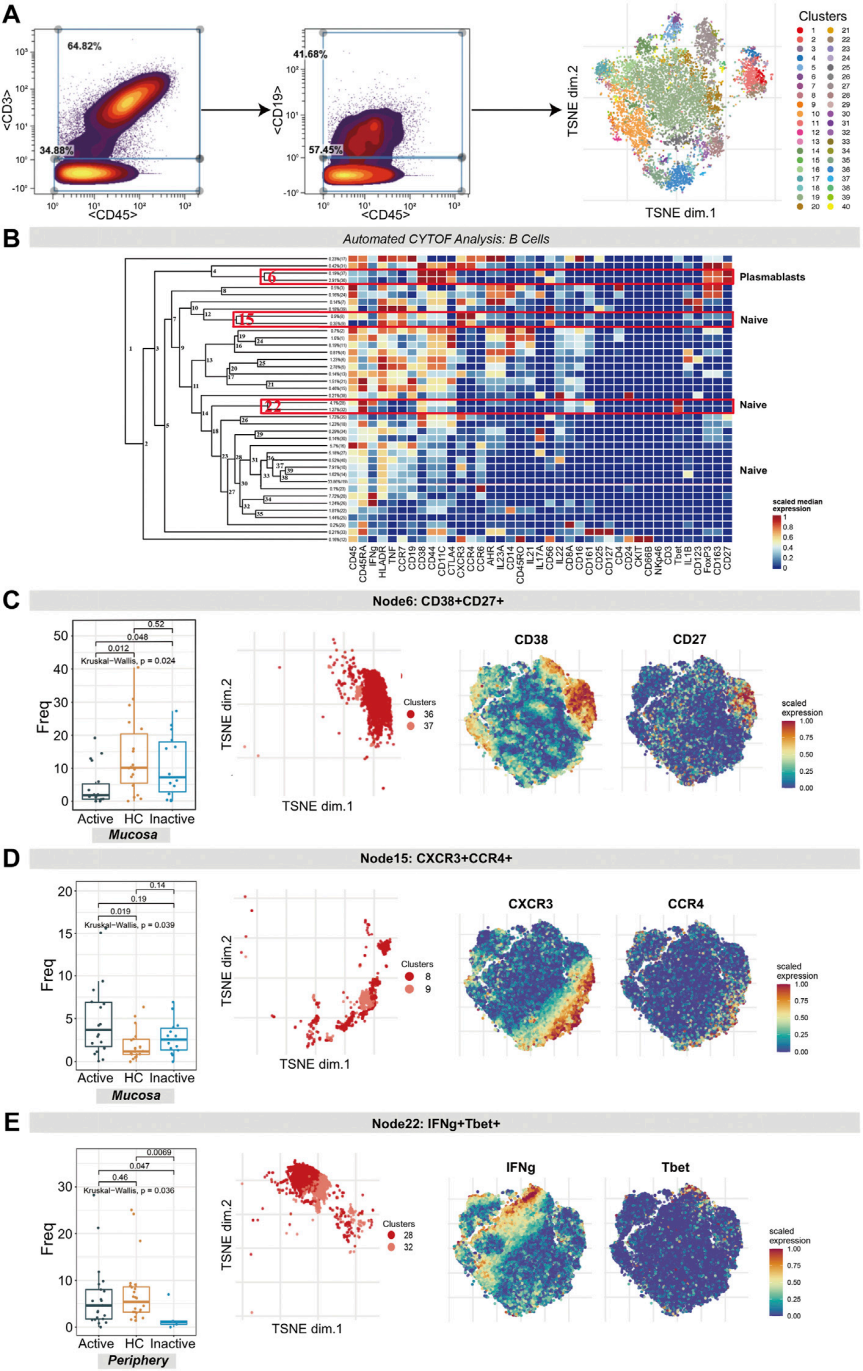
CyTOF analysis differentiated naïve B cell and plasmablast populations in UC patients and HCs. **(A)** Gating steps (left) and t-SNE plot (right) of selected B cells. **(B)** Cluster dendrogram, heatmap and selected nodes of B cells. **(C–E)** Abundance boxplots and t-SNE plots of node 6 **(C)**, node 15 **(D)** and node 22 **(E)** in B cells, with respective selected marker heatmaps for reference.

CD3^−^CD45^+^CD19^+^ B cells were clustered in an analysis dedicated to exploring the differences in B cell subset populations among the UCa, UCin and HC groups (Figure 3A,B). Multiple B cell subsets were categorized, including CD38^+^CD27^+^ plasmablasts, CD27^−^ naïve cells, and CD27^+^ memory cells.

The difference in the number of plasmablasts (node 6 in Figure 3B, containing clusters 37 and 36) among the three groups was statistically significant (*p* = 0.024). A decreasing trend of these plasmablasts in UCa mucosa samples was discovered (Figure 3C). In addition, for node 15 (clusters 8 and 9) and node 22 (clusters 28 and 32), the differences in naïve B cells were statistically significant (*p* = 0.039 and *p* = 0.036, respectively). CXCR3^+^CCR4^+^ naïve B cell clusters (node 15), expressing HLA-DR, TNF and CCR7, were found to be expanded more in UCa mucosa than in HC mucosa (*p* < 0.05, Figure 3D). Node 22 (expressing TBET, CD38, CD161 and IFNG) was decreased in UCin peripheral blood compared with HC and UCa peripheral blood (*p* < 0.05, Figure 3E).

### Innate Immune Cell Signatures Differentiate Active Ulcerative Colitis From Inactive Ulcerative Colitis in Mucosa and Peripheral Blood

**FIGURE 4:**
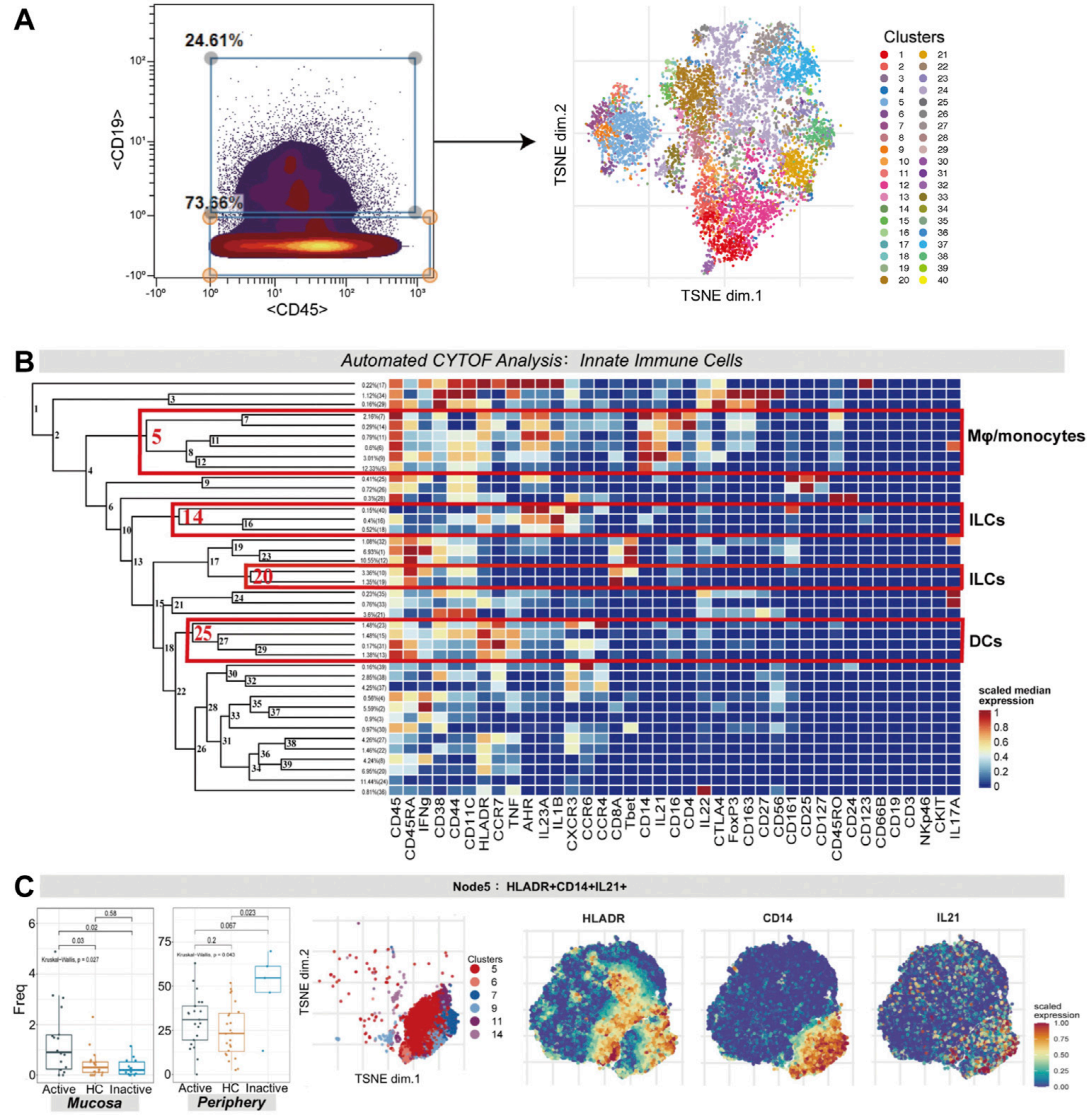
**(A)** CyTOF analysis showed the enrichment of HLA-DR^+^CD14^+^IL21^+^ macrophages/monocytes in UC mucosa. Gating steps (left) and t-SNE plot (right) of selected innate immune cells. **(B)** Cluster dendrogram, heatmap and selected nodes of innate immune cells. **(C)** Abundance boxplot and t-SNE plot of node 5 with HLADR/CD14/IL21 marker heatmaps for reference.

Innate immune cells (CD3^−^CD45^+^CD19^−^ cells) were manually gated with CyTOF (Figure 4A). Several innate immune cell subsets were identified according to their high expression factors, mainly macrophages/monocytes (CD11C^+^CD14^+^), dendritic cells (DCs; HLA-DR^+^CD11C^+^CD14^−^), and innate lymphocytes (ILCs; HLA-DR^-^IFNG^+^CD161^+^CD127^+/−^) (Figure 4B).

There were statistically significant differences in the abundance levels of HLA-DR^+^IL21^+^ macrophages/monocytes among the three groups in both the mucosa (*p* = 0.027) and peripheral blood samples (*p* = 0.043). HLA-DR^+^IL21^+^ macrophages/monocytes (node 5, covering clusters 5, 6, 7, 9, 11, and 14) were enhanced in UCa mucosa (*p* < 0.05, Figure 4C). Nevertheless, in peripheral blood samples, these cells were enriched the most in the UCin group in contrast to the UCa and HC groups (*p* < 0.05, Figure 4C).

**FIGURE 5:**
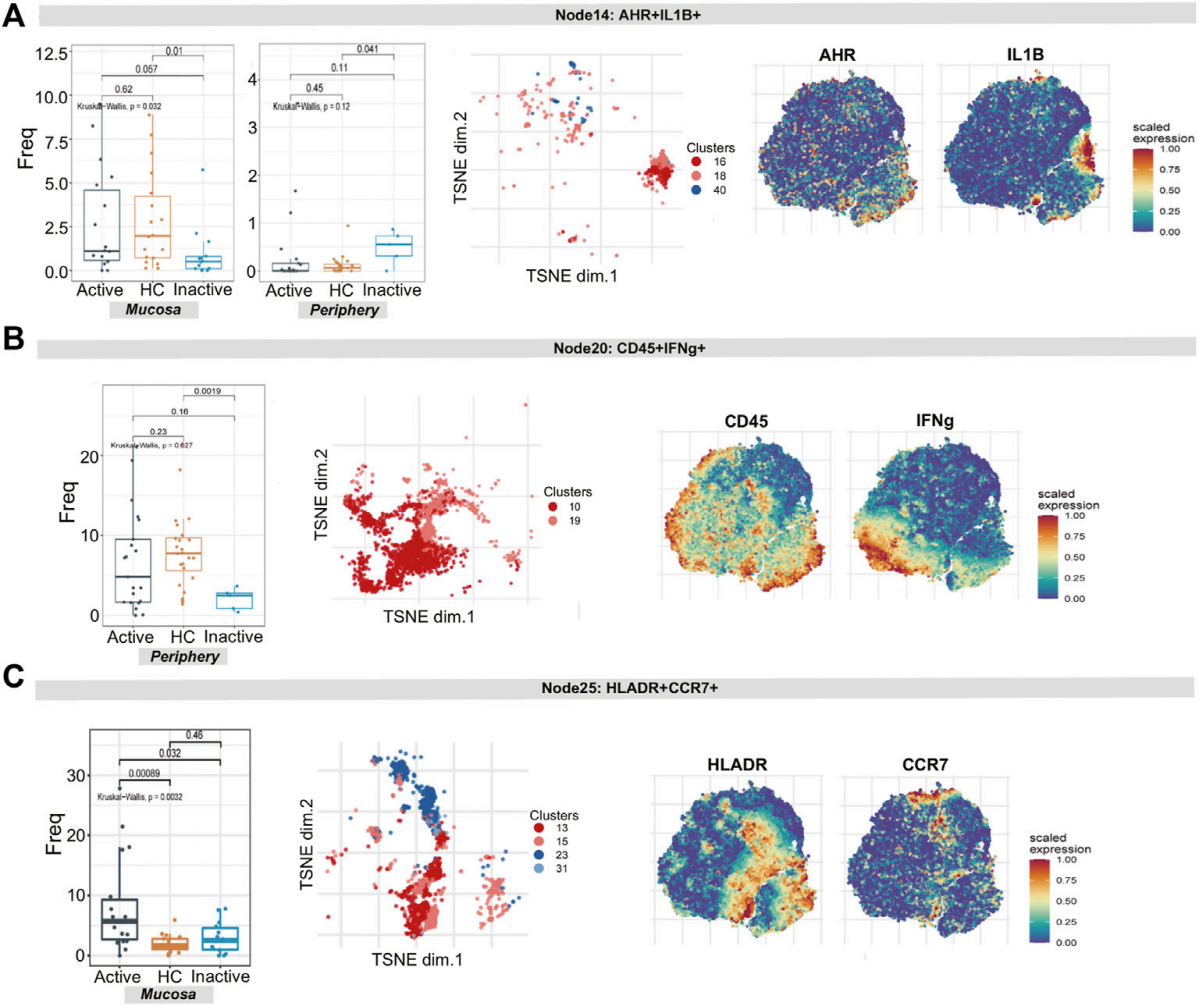
CyTOF demonstrates differential innate lymphoid cell and dendritic cell signatures in UC patients and HCs. **(A–C)** Abundance boxplots and t-SNE plots of node 14 **(A)**, node 17 **(B)** and node 18 **(C)** in innate immune cells, with respective selected marker heatmaps for reference.

For node 14 (a cluster of ILCs), there was a distinction in the cell frequency among the three groups in the mucosa sample (*p* = 0.032). Node 14, which expressed AHR, IL1B, CXCR3 and IL23A, was specifically reduced in UCin mucosa (*p* < 0.05, Figure 5A). Although there was a higher abundance of AHR^+^IL1B^+^ ILCs (node 14) in UCin peripheral blood, the difference was not statistically significant (*p* = 0.12, Figure 5A). There was a decrease in CD45^+^IFNG^+^ ILCs (node 20) in UC peripheral blood compared to the levels in HC peripheral blood (*p* < 0.05, Figure 5B), but none of these differences were statistically significant in mucosa.

For node 25 (clusters 13, 15, 23, and 31), the differences in the number of HLA-DR^+^CCR7^+^ DCs among the three groups were statistically significant (*p* = 0.0032). In detail, these cells were increased more in UCa mucosa than in UCin and HC mucosa (*p* < 0.05, Figure 5C).

### Tolerant NK Cells and Regulatory NK Cells Were Diminished While Cytotoxic NK Cells Were Enriched in Active Ulcerative Colitis Mucosa

**FIGURE 6:**
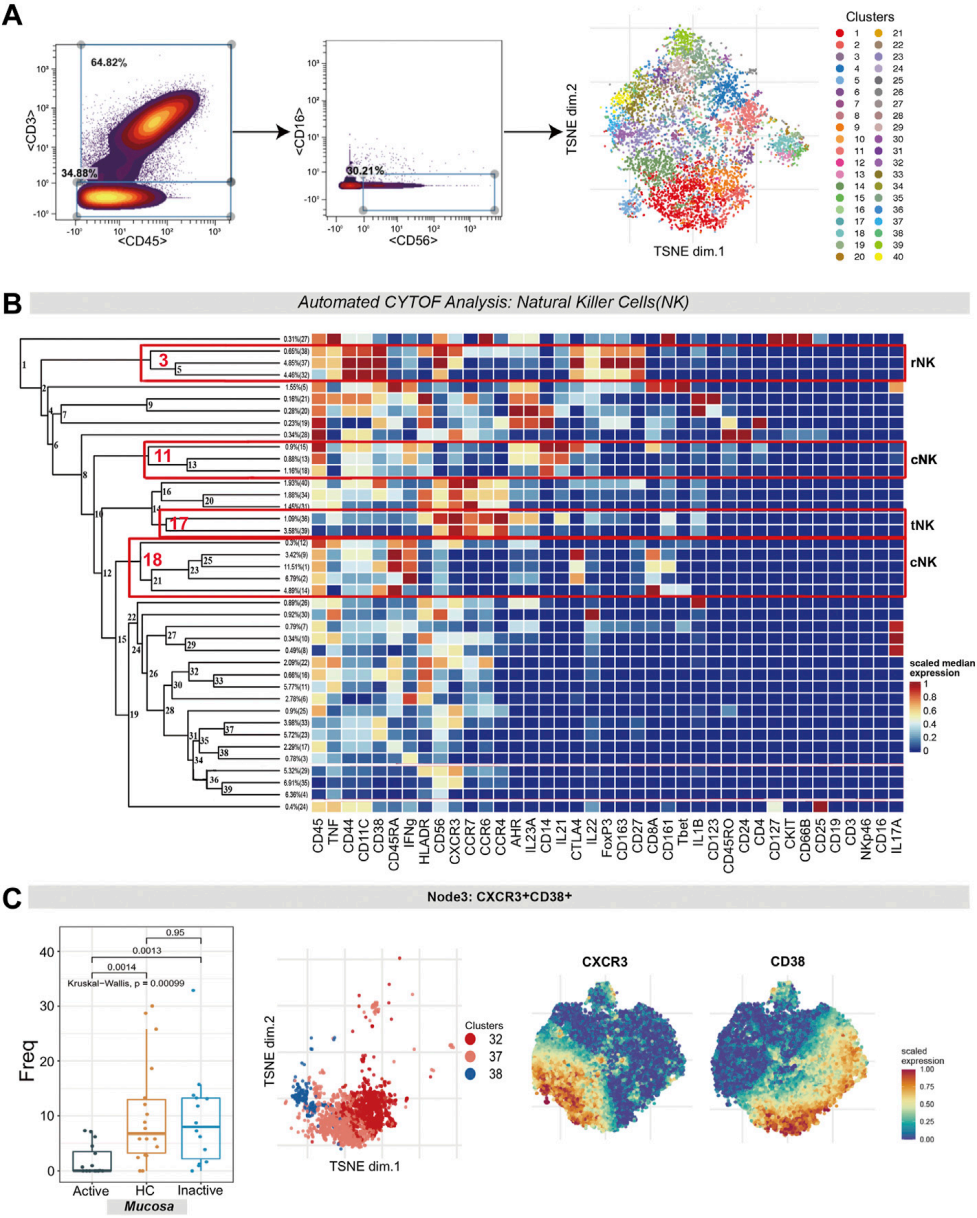
Natural killer cell analysis pointed out the contraction of CXCR3^+^CD38^+^ regulatory NK cells in UC mucosa. **(A)** Gating steps (left) and t-SNE plot (right) of selected NK cells. **(B)** Cluster dendrogram, heatmap and selected nodes of NK cells. **(C)** Abundance boxplot and t-SNE plot of node 3 with CXCR/CD38 marker heatmaps for reference.

Natural killer (NK) cells (CD45^+^CD3^−^CD56^+^CD16^−^ cells) were identified by manual gating (Figure 6A). Several NK cell subsets were identified according to their high expression factors (Figure 6B), mainly including regulatory NK cells (rNK, CD11^+^CD27^+^), tolerant NK cells (tNK, CD11^−^CD27^−^) and cytotoxic NK cells (cNK, CD11^+^CD27^−^) (Fu et al., 2014).

**FIGURE 7:**
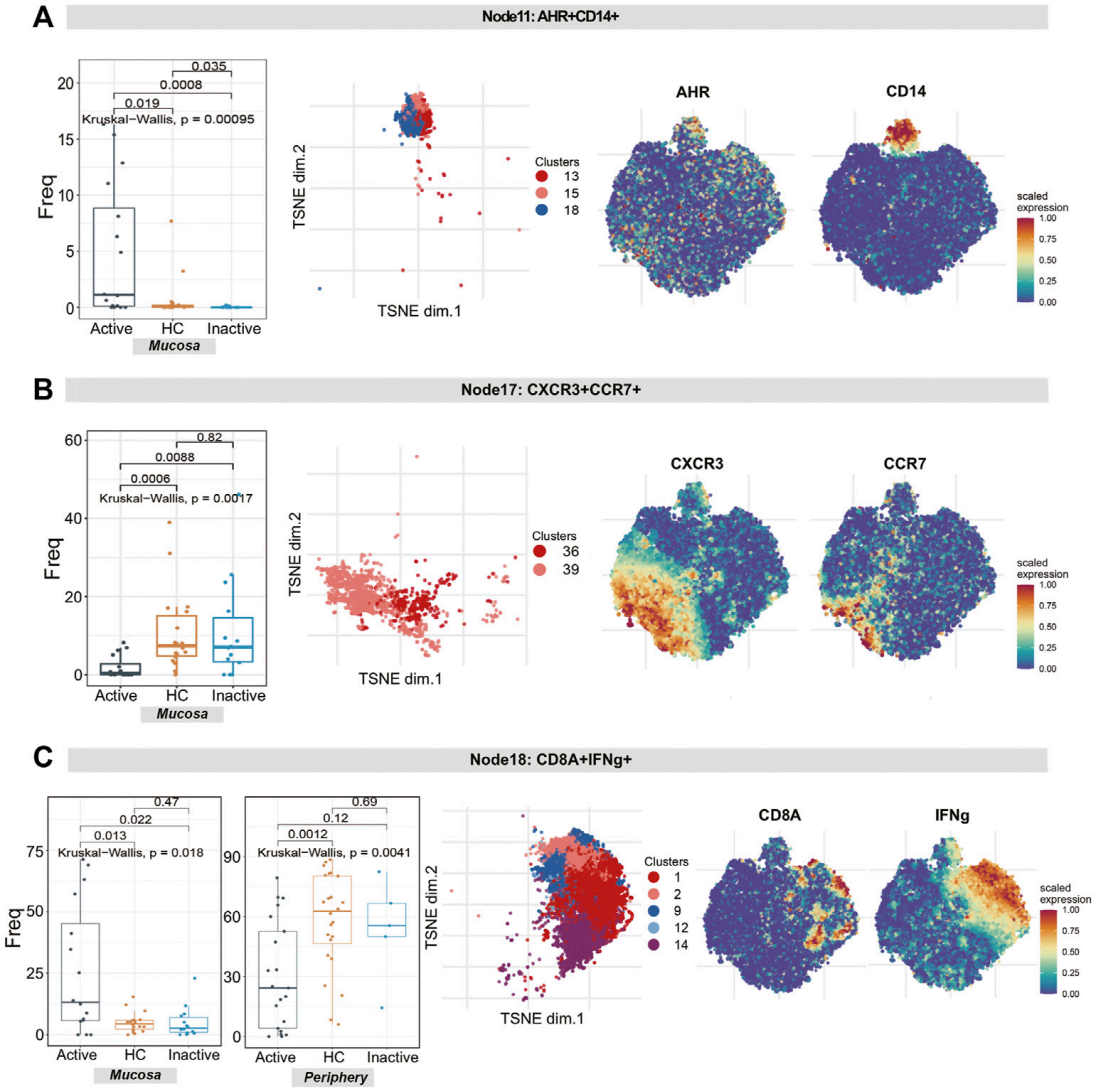
Natural killer cell analysis revealed the diversity of cytotoxic NK cells and tolerant NK cells in UC patients and HCs. **(A–C)** Abundance boxplots and t-SNE plots of node 11 **(A)**, node 17 **(B)** and node 18 **(C)** in NK cells, with respective selected marker heatmaps for reference.

1) The differences in the level of rNK cells (node 3, containing clusters 32, 37 and 38) were statistically significant (*p* = 0.00099). Specifically, rNK cells expressing CD38, CXCR3, CTLA4, IL22 and TNF were diminished in UCa mucosa compared to HC and UCin mucosa (*p* < 0.05, Figure 6C). 2) For node 17 (clusters 36 and 39), CXCR3^+^CCR4^+^ tNK cells among three groups were significantly different (*P* = 0.0017), and there was a lower level in UCa mucosa than in HC and UCin mucosa (*p* < 0.05, Figure 7E). (3) For AHR^+^CD14^+^ cNK cells (node 11, covering clusters 13, 15 and 18), there was a significant difference among the three groups (*p* = 0.00095). AHR^+^CD14^+^ cNK clusters were prominently increased in UCa mucosa (*p* < 0.05, Figure 7D). In addition, there were statistically significant differences in the abundance level of node 18 among the three groups in either mucosa (*p* = 0.018) or peripheral blood samples (*p* = 0.0041). IFNG^+^CD8A^+^CTLA4^+^ cNK cells (node 18, including clusters 1, 2, 9, 12, and 14) were significantly expanded in UCa mucosa and conversely declined in UCa peripheral blood compared with levels in HC samples (*p* < 0.05, Figure 7F).

### Single-Cell Analysis Distinguished and Validated the Differences Among Active Ulcerative Colitis, Inactive Ulcerative Colitis and Healthy Controls in Mucosa and Peripheral Blood

Finally, intestinal mucosal biopsy samples, including 2 UCa patients, 2 UCin patients and 2 HC patients, were obtained (our dataset). scRNA-seq on mucosal samples from 6 subjects was performed to validate the previous findings. We identified 5 immune cell lineages (including NK cells, T cells, B cells, DCs, and mast cells) and 4 nonimmune cell lineages (including epithelial cells, endothelial cells, neuroepithelial cells, smooth muscle cells and tissue muscle cells, Supplementary Figure S1).

GSE125527 and GSE116222 were also used to validate the results of CyTOF. A total of 30 samples, including 16 HC samples and 14 UC samples, were included in GSE125527. For GSE125527, we identified 5 immune cell lineages, including NK cells, T cells, B cells, DCs, and macrophages (Supplementary Figure S2). A total of 9 samples, including 3 HC samples, 3 UCa samples and 3 UCin samples, were included in GSE116222. For GSE11622, we identified 5 immune cell lineages (including NK cells, T cells, B cells, DCs, and mast cells) and 2 nonimmune cell lineages (including epithelial cells and neuroepithelial cells, Supplementary Figure S3).

**FIGURE 8:**
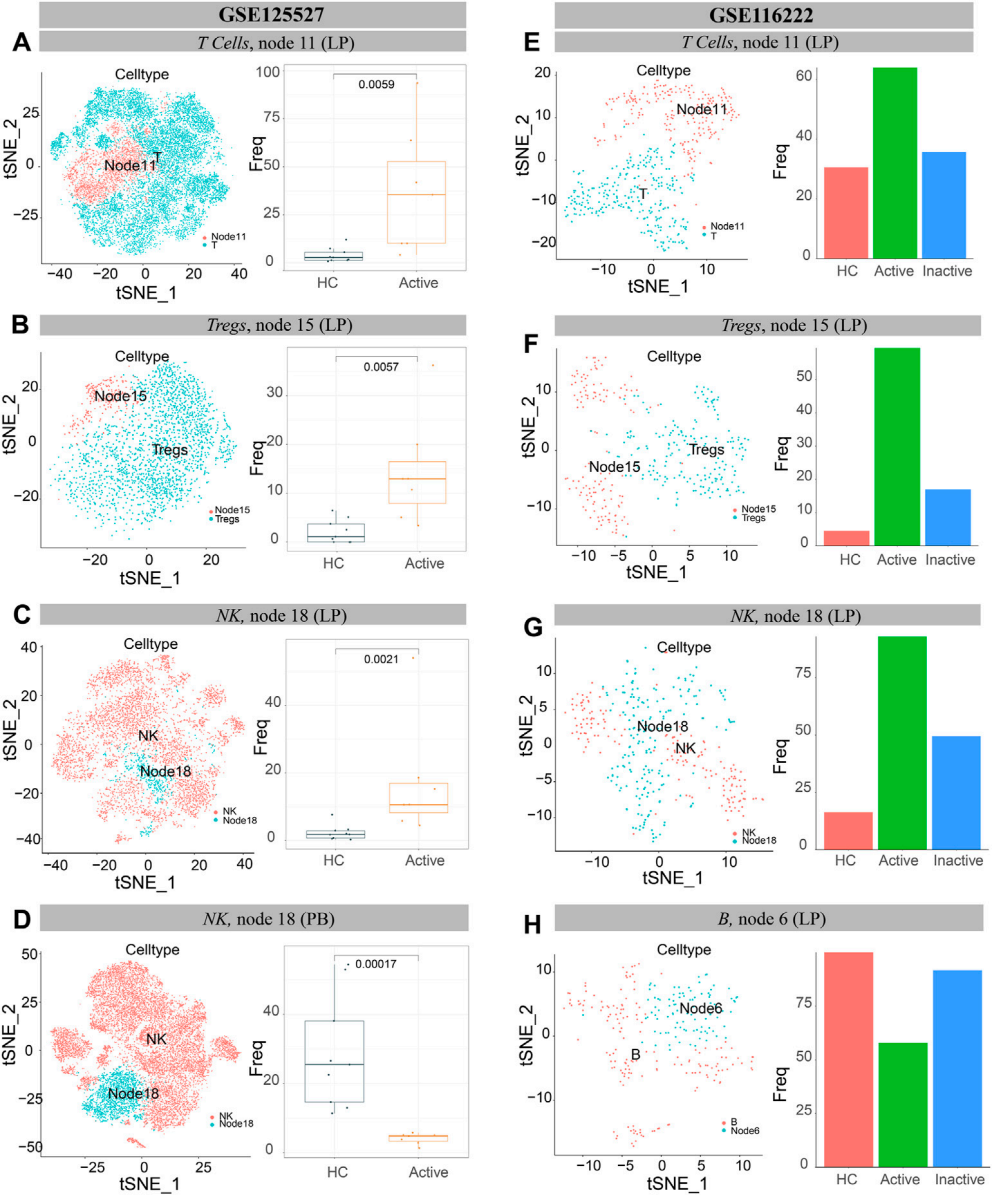
Verification of single cells derived from UC and healthy samples. **(A–D)** t-SNE plots of T lymphocytes **(A)**, Tregs cells **(B)** and NK cells **(C,D)** from all participants across mucosa (LP) and peripheral blood (PB) in GSE125527. “Node” labels in the t-SNE plots indicate the same subclusters as the nodes in CyTOF, and the labels “T” **(A)**, “Tregs” **(B)** and “NK” **(C,D)** indicate individual cell populations (left). Boxplot of the selected T lymphocyte **(A)**, Treg cell **(B)** and NK cell **(C,D)** cluster abundances in mucosa (LP) and peripheral blood (PB) samples of HCs and UCa patients (right), with Wilcoxon rank-sum test results; statistical significance was set at *p* < 0.05. **(E–H)** t-SNE plots of T lymphocytes **(E)**, Treg cells **(F)**, NK cells **(G)** and B cells **(H)** from all participants’ mucosa (LP) in GSE116222. “Node” labels indicate subclusters with nodes in CyTOF, and “T” **(E)**, “Tregs” **(F)**, “NK” **(G)** and “B” **(H)** labels indicate respective cell populations (left). Bar charts of the selected T lymphocyte **(E)**, Treg cell **(F)**, NK cell **(G)** and B cell **(H)** cluster abundances in mucosa (LP) samples of HCs, UCa patients and UCin patients (right).

**FIGURE 9:**
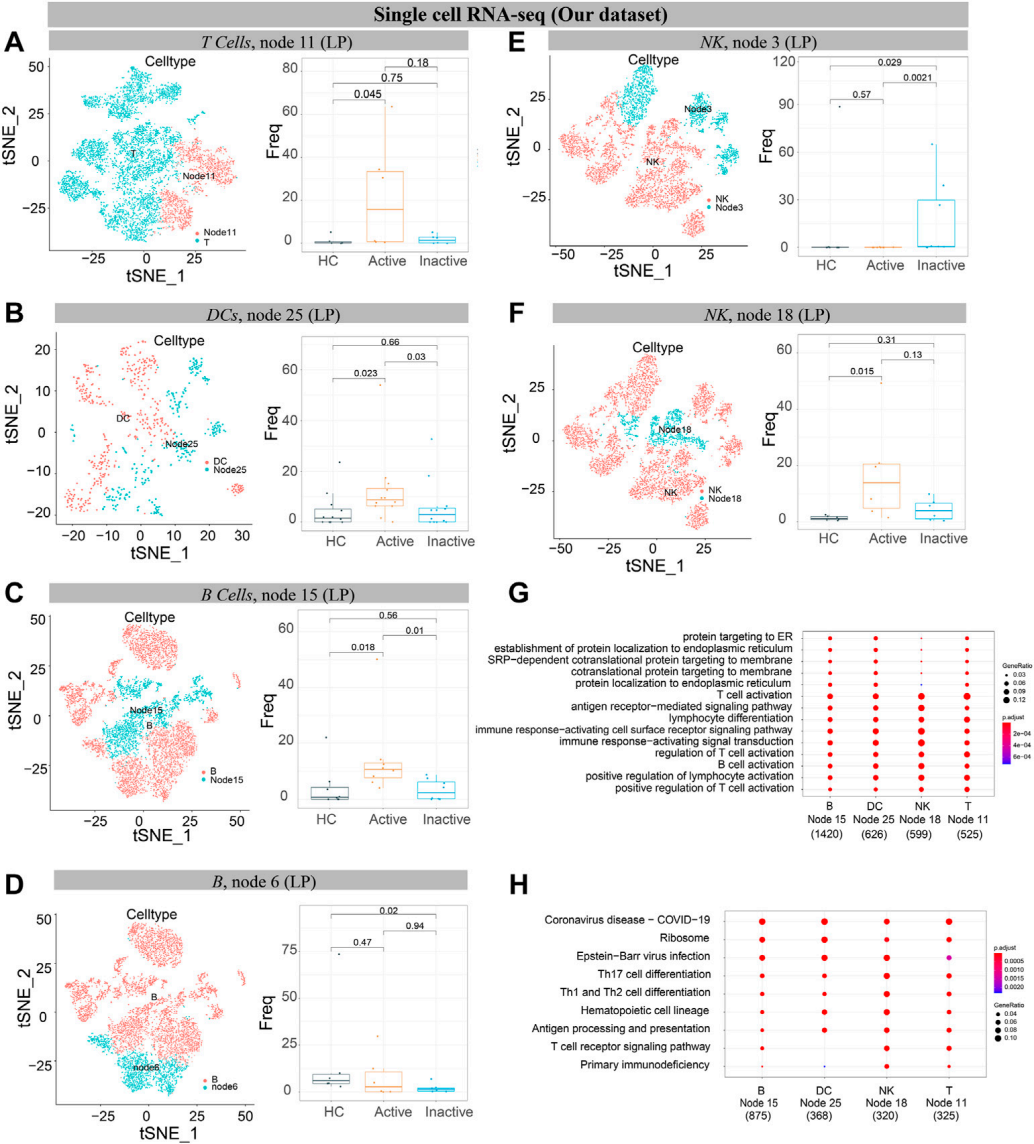
Verification of single cells derived from UC and healthy samples. **(A–F)** t-SNE plots of T cells **(A)**, DCs **(B)**, B cells **(C, D)**, and NK cells **(E, F)** from 6 participants’ mucosa (LP) samples (our dataset). “Node” labels in t-SNE plots indicate the same subclusters as nodes in CyTOF (left). Boxplot of selected T cell **(A)**, DC **(B)**, B cell **(C,D)**, and NK cell **(E,F)** cluster abundances in mucosa (LP) samples of HC, UCa and UCin (right), with Wilcoxon rank-sum test results; statistical significance was set at *p* < 0.05. **(G)** The GO enrichment analysis. **(H)** The top 10 signaling pathways according to KEGG analysis.

The results of GSE125527 and our dataset showed that CCR6^+^TNF^+^CD161^+^ EM T cells (node 11) were enriched in UCa mucosa, and the abundance levels between the UCa and HC groups were significantly different (*p* < 0.05, Figures 8A, 9A). In addition, node 11 had a comparatively increased trend in UCa mucosa compared to UCin and HC mucosa (Figure 8E). For CD38^+^TNF^+^ Tregs (node 15), the abundance in UCa was significantly higher than that in HC (*p* < 0.05, Figure 8B). Similarly, there was a trend in which node 15 was highest abundant in UCa mucosa and lowest abundant in HC mucosa (Figure 8F).

Single-cell analysis of three datasets verified that IFNG^+^CD8A^+^CTLA4^+^ cNK cells (node 18) were obviously increased in UCa mucosa and diminished in UCa peripheral blood (*p* < 0.05, Figures 8C,D, 9F). Moreover, an enrichment in cNK cells (node 18) in UCa in contrast with both HC and UCin mucosa was confirmed (Figure 8G). Increased abundance of CXCR3^+^CD38^+^ rNK cells (node 3) in UCin was also observed, and the difference between UCin and the other two groups was statistically significant (*p* < 0.05, Figure 9E).

The results for B cells demonstrated that the abundance of node 6 (CD38^+^CD27^+^ plasmablasts) was the highest in HC mucosa (Figures 8H, 9D), and the abundance levels between HC and UCin mucosa were significantly different (*p* < 0.05). In addition, scRNA-seq found that there was significantly higher abundance of node 15 (CXCR3^+^CCR4^+^ naïve B cells) in UCa mucosa than in HC and UCin mucosa (*p* < 0.05, Figure 9C).

HLA-DR^+^CCR7^+^ DCs (innate node 25) measured by scRNA-seq among the three groups showed the same trend as the results with CyTOF, and the abundance of DCs in UCa mucosa was greater than that of the HC and UCin groups (*p* < 0.05, Figure 9B).

### Differentially Expressed Gene-GO/KEGG Analysis to Investigate Potential Molecular Regulators of UC and to Identify the Function of Candidate Marker Nodes in UC

CCR6^+^TNF^+^CD161^+^ T cells (T node 11), HLA-DR^+^CCR7^+^ DCs (innate node 25), CXCR3^+^CCR4^+^ B cells (B node 15), and CD8A^+^IFNG^+^ NK cells (NK node 18), which were increased in the UCa group and decreased in the HC group, were selected for gene-GO/KEGG analysis (Figures 9G,H).

GO analysis indicated that the primary functions of these four subsets were similar. It revealed the activation and differentiation of many immune cells in UC, including T cell activation, B cell activation, regulation of T cell activation, and lymphocyte differentiation. These subsets also revealed a higher proportion of biological processes, consisting of positive regulation, response to stress, cellular response, and cell adhesion.

KEGG pathway analysis also demonstrated disease enrichment in each subset, including coronavirus disease (COVID-19) and rheumatoid arthritis.

In Supplementary Figure S4, at a 10% FDR, we identified 6635 DA neighbourhoods. As shown in the figure (Supplementary Figure S4C), immune cells were differentially expressed in UC and HC conditions, which is consistent with the previous results.

## Discussion

The etiopathogenesis of UC is not fully understood, but immune-mediated mechanisms might be responsible for dysregulated immune responses against intraluminal antigens in genetically predisposed individuals (Zhang et al., 2017). Recent studies have confirmed that some biologics, such as antitumor necrosis factor (TNF), could maintain remission in patients with UC and improve the long-term prognosis of UC (Naganuma et al., 2013; Moss 2014). Therefore, CyTOF and single-cell analysis were utilized to determine the specific immune characteristics of UCa and UCin.

Novel subgroups were identified according to CyTOF and scRNA-seq analysis, including IFNG^+^TNF^+^IL-17A^+^CD161^+^ EM T cells, TNF^+^IL-17A^++^ EM Tregs, CXCR3^+^CTLA4^+^ EM Tregs, CXCR3^+^CCR4^+^TNF^+^ naïve B cells, HLA-DR^+^CCR7^+^TNF^+^ DCs, CD14^++^IL21^+^CD16^+^IL23A^+^AHR^+^ macrophages/monocytes, CD38^++^CD44^++^TNF^+^IL22^+^CXCR3^+^ tNK cells and CXCR3^++^CCR7^+^CCR6^+^CCR4^+^ tNK cells.

In our findings, the frequency of IFNG^+^TNF^+^ EM T cells (node 11) in UCa mucosa was significantly higher than those in UCin and HC mucosa. These results are in accord with recent studies. Lovisa S et al. indicated that with microorganisms damaging the gut, a large number of EM T cells gather and produce proinflammatory factors to defend against these microorganisms (Lovisa et al., 2019). We inferred that these cells can be used as diagnostic targets to distinguish UCa from UCin.

We found that some clusters of Treg cells were enriched in UC patients. TNF^+^IL-17A^++^ EM Tregs (node 15) and CXCR3^+^CTLA4^+^ EM Tregs (node 27) were markedly increased in UCa or UCin patients compared with HCs, which was consistent with previously published studies (Kamikozuru et al., 2009; Li et al., 2017; Sun et al., 2017; Ueno et al., 2018; Long et al., 2020b). 1) Tregs are known to be a key subset of cells that play an inhibitory role in maintaining immune homeostasis. The key role of dysregulated Treg responses in the propagation and perpetuation of intestinal inflammation is widely accepted (Raphael et al., 2015). 2) In our study, the abundance of CTLA4, IL-17A and TNF in Tregs (node 15) was increased in UCa mucosa, and CTLA4, CXCR3 and CD38 in Tregs (node 27) were specifically increased in UCin peripheral blood. Some marker genes play a vital role in Treg cell function. For example, CTLA4 is an important negative regulator of the immune system. Ovcinnikovs V et al. indicated that CTLA4 expressed on Tregs could dynamically regulate the phenotype of DC trafficking to sites of inflammation from peripheral tissues (Ovcinnikovs et al., 2019). Enhancement of DC stimulation and overresponse to bacterial antigens trigger mucosal damage (Targan and Karp 2005; Tatiya-Aphiradee et al., 2018).

CXCR3 is a chemokine receptor in the CXC family that has been implicated in the pathogenesis of UC. Previous data have shown that CXCR3 expression in peripheral Tregs inhibits the immune response and systemic inflammatory cytokines, thereby reducing inflammation and leading to effective inhibition of colitis (Abron et al., 2018).

Our findings showed that IFNG^+^ NK cells (node 18) were enriched in UC patients’ mucosa but decreased in their peripheral blood. Possible mechanisms or reasons include the following: 1) Some studies have indicated that IFNG^+^ NK cells play an important role in the tissue inflammation associated with UC by activating effector immune cells and enhancing antigen presentation (Hisamatsu et al., 1996; El Bougrini et al., 2006). Upon IFNG binding, the IFNGR1 intracellular domain opens to allow association of the downstream signaling components JAK2, JAK1 and STAT1, leading to STAT1 activation, nuclear translocation and transcription of IFNG-regulated genes (Greenlund et al., 1993). 2) Some proinflammatory cytokines (IL-21 and IL-23) could potently induce IFNG^+^ NK cell activation to secrete high levels of proinflammatory cytokines and promote cytolytic activities against target cells in the mucosa (Yadav et al., 2011). 3) Prior research reported that in UCa, peripheral NK cells were decreased significantly compared to those in UCin (Wang et al., 2017). This might be due to the role of NK cells in the inflammatory environment. In response to intestinal bacterial infections, NK cells can produce IFNG. Then, IFNG stimulates the recruitment of additional NK cells from peripheral blood, and this process leads to expansion of the antibacterial immune response (Poggi et al., 2019). It could be speculated that these immune cell subsets might be therapeutic targets for UC.

Immune population differences distinguishing UCa from UCin were primarily found in DC cells. Concomitant with colitis development, DCs in inflamed patients increase in number and upregulate the expression of CCR7, TNF, and CXCR3 costimulator molecules. These cells in mucosa are antigen-presenting cells that can influence the differentiation of cytokine responses and induce proinflammatory cytokine responses in T cells. Such interactions may be critical to the course of UC (Drakes et al., 2005; Baumgart et al., 2011). Hence, it is hypothesized that in the UC colon, CXCR3^+^ DC-T cell interactions may create conditions with an abundance of proinflammatory cytokines such as TNF, which favors the inflammatory state.

Our results showed that CXCR3 was highly expressed in many immune cell subsets: HLA-DR^+^CCR7^+^CXCR3^+^ DCs (node 25), CXCR3^++^CCR4^++^TNF^+^CCR7^+^ B cells (node 15), CXCR3^+^CTLA4^+^ EM Tregs (node 27), CXCR3^+^CD38^++^TNF^+^IL22^+^ rNK cells (node 3) and CXCR3^++^CCR7^+^CCR6^+^CCR4^+^ tNK cells (node 17). CXCR3 is a chemokine receptor in the CXC family that has been implicated in the pathogenesis of UC. Singh UP et al. indicated that CXCR3 was upregulated at sites of experimental colitis (Singh et al., 2016). CXCR3 plays a pivotal role in controlling the migration of disease-inducing CD4^+^CD25^+^ T cells into the intestinal wall (Kristensen et al., 2006).

The inflamed mucosa of inflammatory bowel disease (IBD) patients shows increased enrichment of CD4^+^CXCR3^+^ T cells (Singh et al., 2007; Wadwa et al., 2016). In the last few years, strong experimental and clinical evidence has been obtained that supports the idea that the CXCR3 pathway is involved in the development of autoimmune diseases, especially by creating local amplification loops of inflammation in target organs, thereby worsening the clinical manifestations (Lacotte et al., 2009).

Several limitations exist in the current study. 1) Some novel cell subsets were not found in the single-cell analysis. In the GSE125527 dataset, there are no inactive UC samples. 2) To determine the pathogenic factors or disease-related biomarkers, further mechanistic research is needed.

## Conclusion

In summary, the results suggest that the novel subsets can be used to distinguish the characteristics among Uca, UCin and HC mucosal and peripheral immune cells. This information might be utilized to develop interventions that focus on specific immune cell subsets in patients with UCa and UCin. The combined application of CyTOF analysis and scRNA-seq analysis broadens the method of research, making it possible to reveal more comprehensively the immune cells and related genes.

## Data Availability

Publicly available datasets were analyzed in this study. This data can be found here: [https://www.ncbi.nlm.nih.gov/geo/query/acc.cgi?acc=GSE125527/GSE125527] and [https://www.ncbi.nlm.nih.gov/geo/query/acc.cgi?acc=GSE116222/GSE116222]. CyTOF files (.fcs files) can be accessed through a publicly available experiment on Cytobank platform (https://premium.cytobank.org) entitled Raw mass cytometry data from: Mitsialis et. al. Single-Cell Analyses of Colon and Blood Reveal Distinct Immune Cell Signatures of Ulcerative Colitis and Crohn’s Disease. Gastroenterology. 2020.
