# Supplementary material for: Mass Cytometry and Single-Cell Transcriptome Analyses Reveal the Immune Cell Characteristics of Ulcerative Colitis
Source: Front Mol Biosci. 2022 Jun 23;9:859645. doi: 10.3389/fmolb.2022.859645 (PMC9260076; doi:10.3389/fmolb.2022.859645)
Supplement: Supplementary file 1 [file DataSheet1.docx]

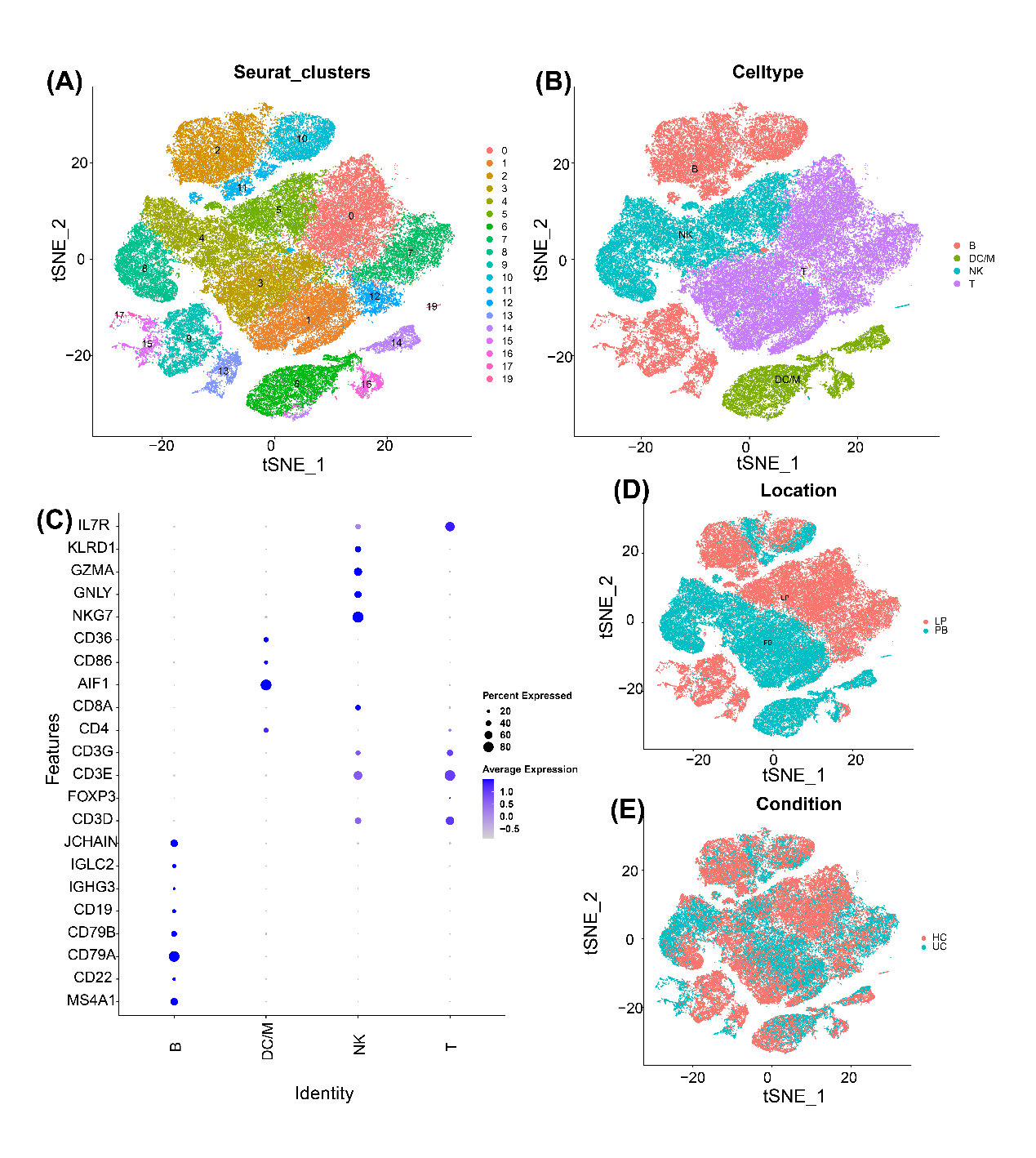


**Supplementary Figure 1. Overview of Single-cell analyses reveal cellular composition of the human immune system in three group.**

(A, B, D, E) t-SNE plot of all the single cells in mucosa, with each color coded for cluster identity (A), eight major cell types (B), sample origin (UCa, UCin or HC) (D), eight major cell types in UCa, UCin and HC respectively (E). (C) Top marker genes of eight major cell types identified in this profile.


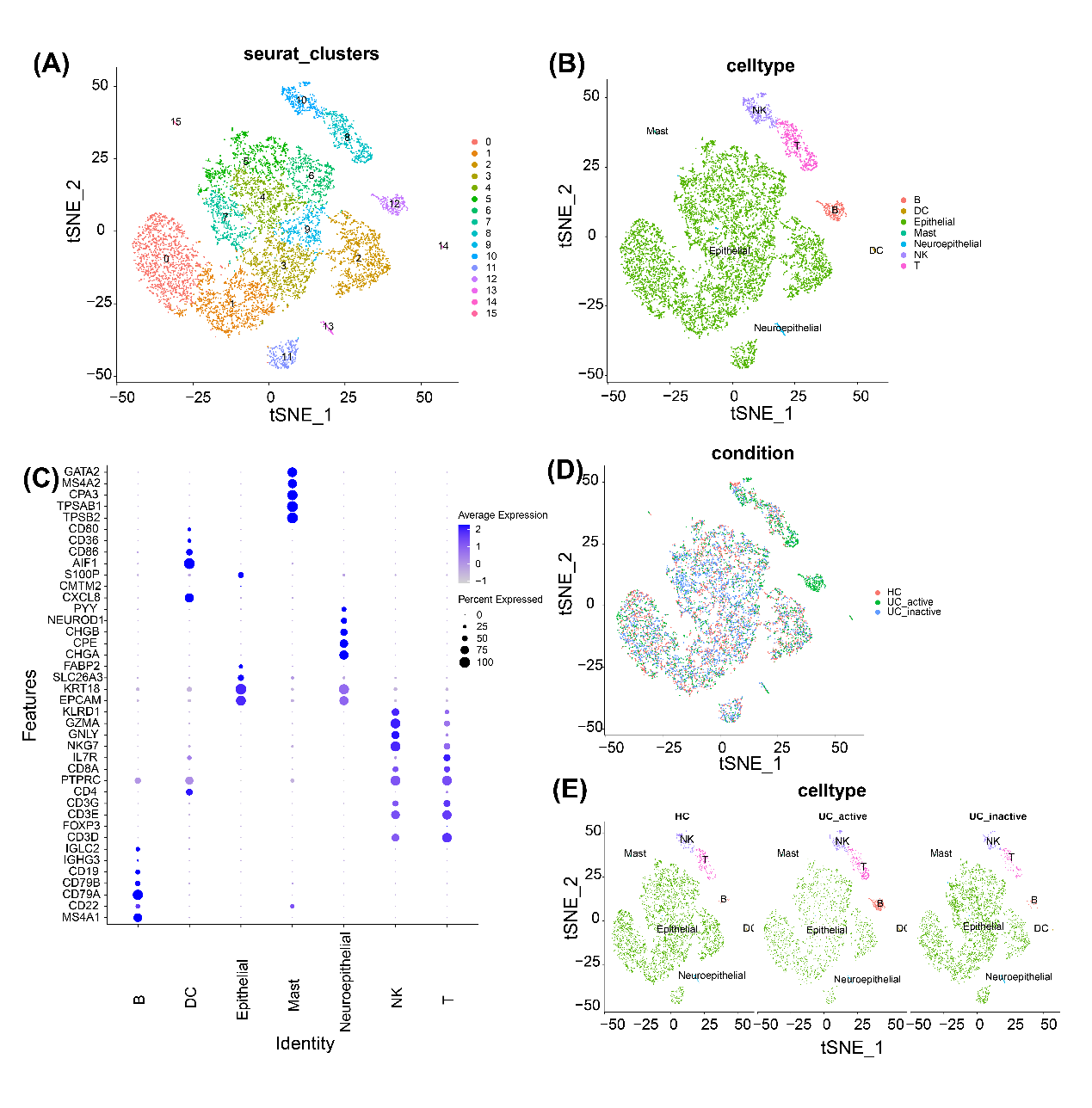


**Supplementary Figure 2. Overview of Single-cell analyses (GSE125527) reveal cellular composition of the human immune system in two group.**

(A, B, D, E) t-SNE plot of all the single cells in GSE125527, with each color coded for cluster identity (A), four major cell types (B), anatomic location (LP: lamina propria or PB: peripheral blood) (D) and sample origin (UC or HC) (E). (C) Top marker genes of four major cell types identified in this profile.


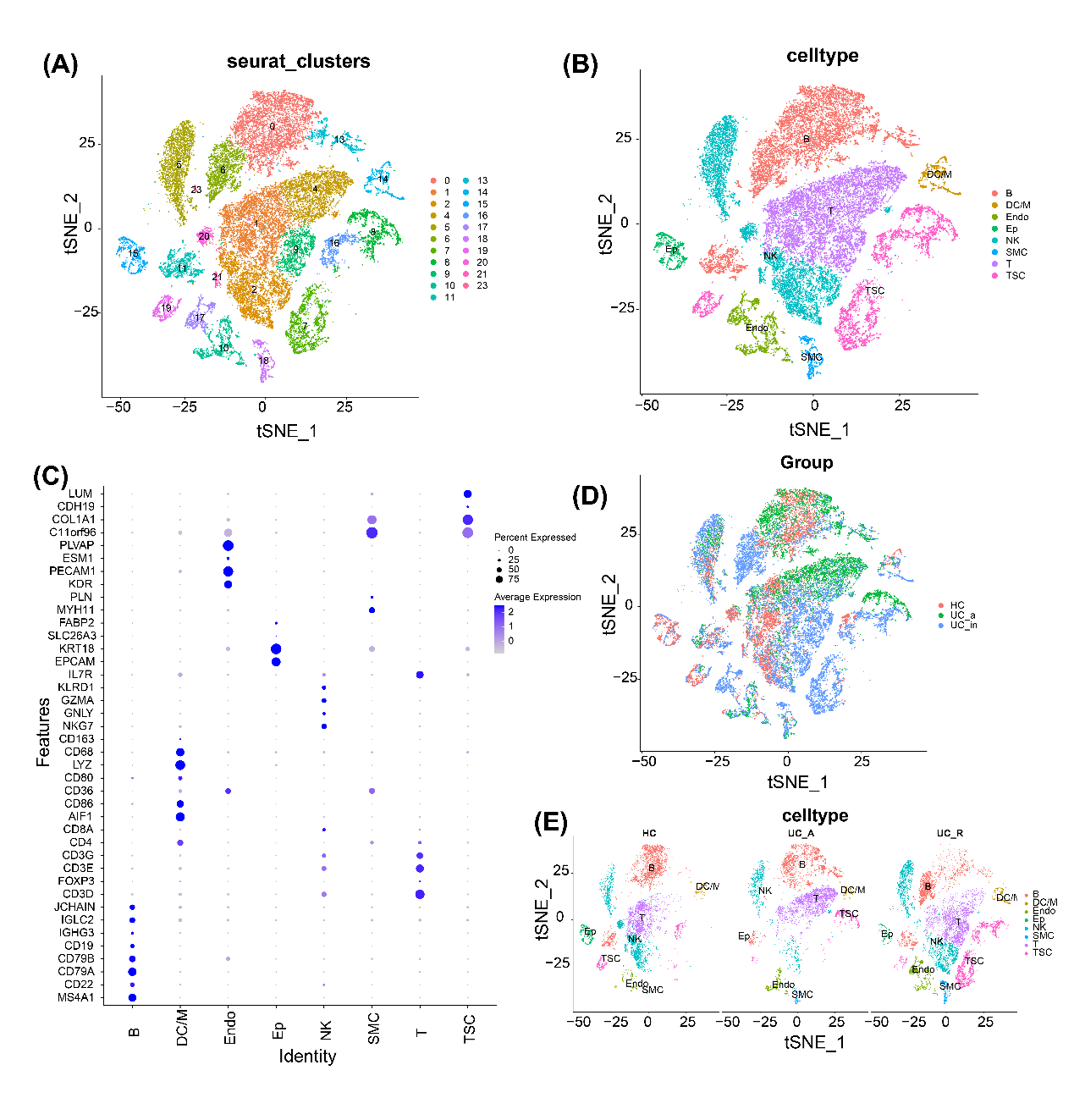


**Supplementary Figure 3. Overview of Single-cell analyses (GSE116222) reveal cellular composition of the human system in three group.**

(A, B, D, E) t-SNE plot of all the single cells in GSE116222, with each color coded for cluster identity (A), seven major cell types (B), sample origin (UCa, UCin or HC) (D), seven major cell types in UCa, UCin and HC respectively (E). (C) Top marker genes of seven major cell types identified in this profile.


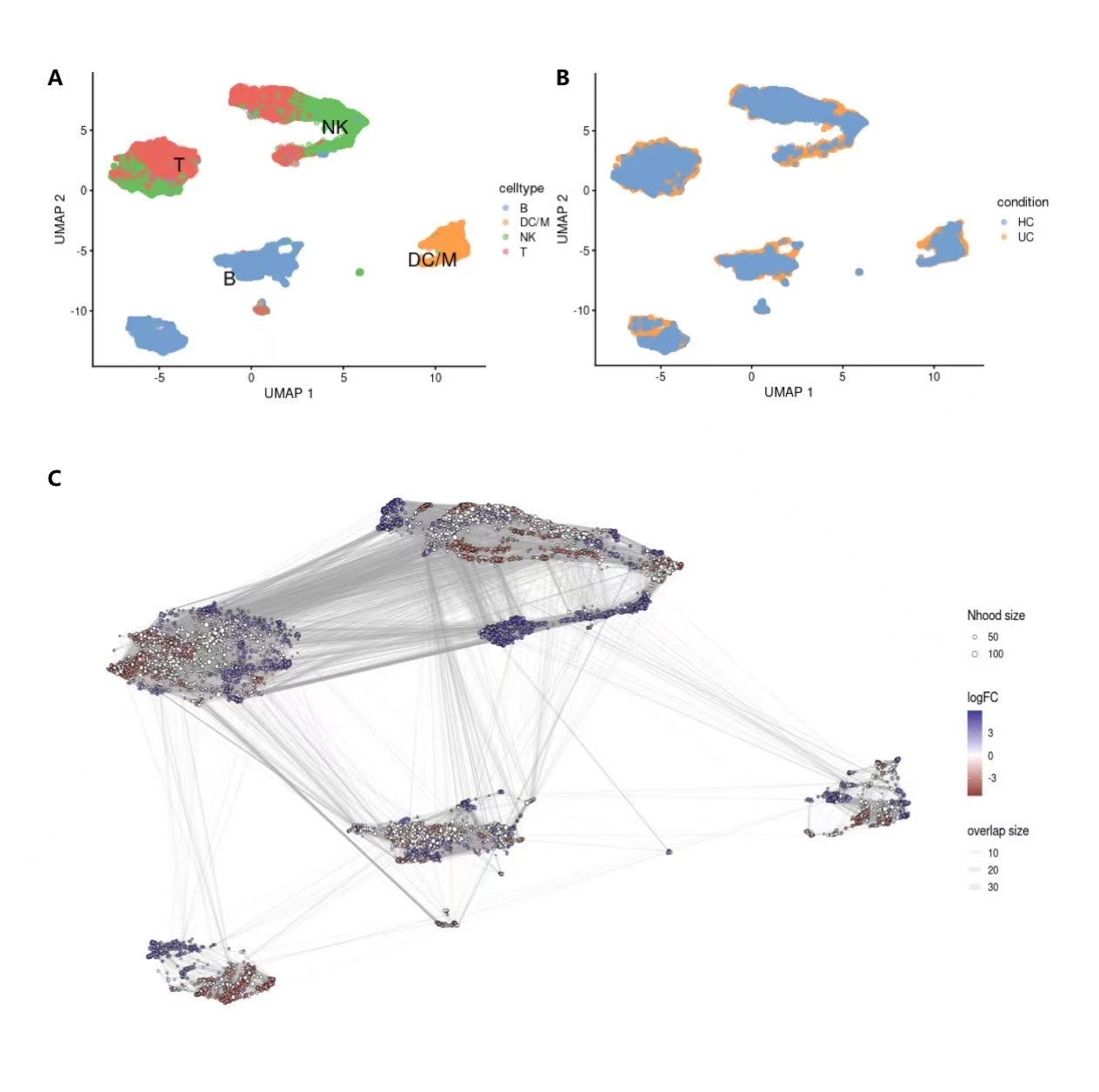


**Supplementary Figure 4. Differential abundance testing with Milo in GSE125527.**

(A-B) A UMAP of single cells sampled from human between UC and HC. (C) A graph representation of the results from Milo differential abundance testing. Each node represents a neighbourhood, while edges indicate how many cells two neighbourhoods have in common. The neighbourhoods displaying significant DA are colored by their log-Fold Change.
